# Silencing of the *CaCP* Gene Delays Salt- and Osmotic-Induced Leaf Senescence in *Capsicum annuum* L.

**DOI:** 10.3390/ijms15058316

**Published:** 2014-05-12

**Authors:** Huai-Juan Xiao, Yan-Xu Yin, Wei-Guo Chai, Zhen-Hui Gong

**Affiliations:** 1 College of Horticulture, Northwest A&F University, Yangling 712100, Shaanxi, China; E-Mails: yuanyi041ban@126.com (H.-J.X.); yinyanxu2008@126.com (Y.-X.Y.); 2 State Key Laboratory of Crop Stress Biology in Arid Areas, Northwest A&F University, Yangling 712100, Shaanxi, China; 3 Institute of Vegetables, Hangzhou Academy of Agricultural Sciences, Hangzhou 311104, Zhejiang, China; E-Mail: kuni@21cn.com

**Keywords:** pepper, cysteine proteinase, leaf senescence, osmotic stresses

## Abstract

Cysteine proteinases have been known to participate in developmental processes and in response to stress in plants. Our present research reported that a novel CP gene, *CaCP*, was involved in leaf senescence in pepper (*Capsicum annuum* L.). The full-length *CaCP* cDNA is comprised of 1316 bp, contains 1044 nucleotides in open reading frame (ORF), and encodes a 347 amino acid protein. The deduced protein belongs to the papain-like cysteine proteases (CPs) superfamily, containing a highly conserved ERFNIN motif, a GCNGG motif and a conserved catalytic triad. This protein localized to the vacuole of plant cells. Real-time quantitative PCR analysis revealed that the expression level of *CaCP* gene was dramatically higher in leaves and flowers than that in roots, stems and fruits. Moreover, *CaCP* transcripts were induced upon during leaf senescence. *CaCP* expression was upregulated by plant hormones, especially salicylic acid. *CaCP* was also significantly induced by abiotic and biotic stress treatments, including high salinity, mannitol and *Phytophthora capsici.* Loss of function of *CaCP* using the virus-induced gene-silencing technique in pepper plants led to enhanced tolerance to salt- and osmotic-induced stress. Taken together, these results suggest that *CaCP* is a senescence-associated gene, which is involved in developmental senescence and regulates salt- and osmotic-induced leaf senescence in pepper.

## 1. Introduction

Leaf senescence is the last stage of leaf development during which the nutrients deposited in leaves are remobilized to other developing organs of the plant [1,2]. However, early leaf senescence resulting from diverse adverse environmental factors leads to the decline of photosynthetic activity, thereby restraining the accumulation of nutrients and limiting crop yield [3]. During leaf senescence, degradation of leaf protein by proteinases provides a large pool of cellular nitrogen for recycling [4]. Proteolysis in plants, involving a number of enzymes, is a complex process [5]. The cysteine proteases (CPs) with a cysteine residue at the active center have been extensively studied because they are involved in a variety of proteolytic functions in higher plants [6].

CPs are ubiquitous in plants. They are associated with many processes of plant development, including seed germination, leaf and flower development, fruit ripening, as well as in legume nodule development [7–12]. For instance, *CsCP* transcripts were enhanced during the development of citrus postharvest peel pitting and *Asnodf32* encodes a nodule-specific CP in *Astragalus sinicus* [10,13]. The silencing of *Asnodf32* via the RNA interference approach delayed root nodule and bacteroid senescence in *Astragalus sinicus.* In addition, CPs are implicated in response to signaling molecules and varying stresses including drought, wounding, as well as pathogen infection [14–18]. The CP enzymes contain two conserved domains: the conserved non-contiguous ERFNIN motif (EX3RX3FX2NX3I/VX3N) is typical for CPs in the Cathepsin L and H-like proteinases, and the GCNGG motif is detectable in all known CPs [19]. Some CPs have a *C*-terminal KDEL motif, which is an endoplasmic reticulum retention signal and involves a programmed cell death (PCD) process [20,21].

Plant genomes encode a lot of CPs, but most senescence-associated CPs belong to the papain family [22,23]. A number of genes encoding papain-like CPs have been isolated from senescing tissues. They exhibit diverse expression patterns during plant developmental stages. The transcripts of some of CP genes are downregulated in senescent leaves such as *Nicotiana tabacum NtCP2* and *Hemerocallis spp SEN102* [24,25]. However, the majority of CP genes are upregulated during leaf senescence. These *CP* genes include *Lycopersicon esculentum SENU2* and *SENU3*, *Nicotiana tabacum NtCP-23* and *NtCP1*, *Arabidopsis SAG2* and *SAG12*, and *Ipomoea batatas SPG31* [25–29]. They are either expressed exclusively during leaf senescence or expression is detectable in young leaves then continues to increase during leaf development. There are a few of senescence-specific CP genes such as *SAG12* in *Arabidopsis* and *NtCP1* in *Nicotiana tabacum*, which are expressed only in senescent leaves [25,27].

In recent years, the research on senescence-related CPs focused on their mRNA levels and post-translational protease activity [8,9,16]. However, the functional property of *CP* is unclear in pepper. Virus-induced gene-silencing (VIGS) technique has been used as an effective tool to identify the function of genes in pepper [30,31]. In this study, we have firstly cloned a novel senescence-associated gene *CaCP* from the pepper plant to analyze its molecular characteristics. Secondly, an attempt was made to identify subcellular location of CaCP protein. Thirdly, the transcripts of *CaCP* were analyzed by quantitative real-time PCR (qRT-PCR). Finally, VIGS was used to analyze the function of the *CaCP* gene. The results demonstrated that *CaCP* may participate in leaf development and be a negative regulation to salt- and osmotic-induced leaf senescence in pepper plants.

## 2. Results and Discussion

### 2.1. Cloning and Sequence Analysis of the CaCP Gene

**Figure 1.**
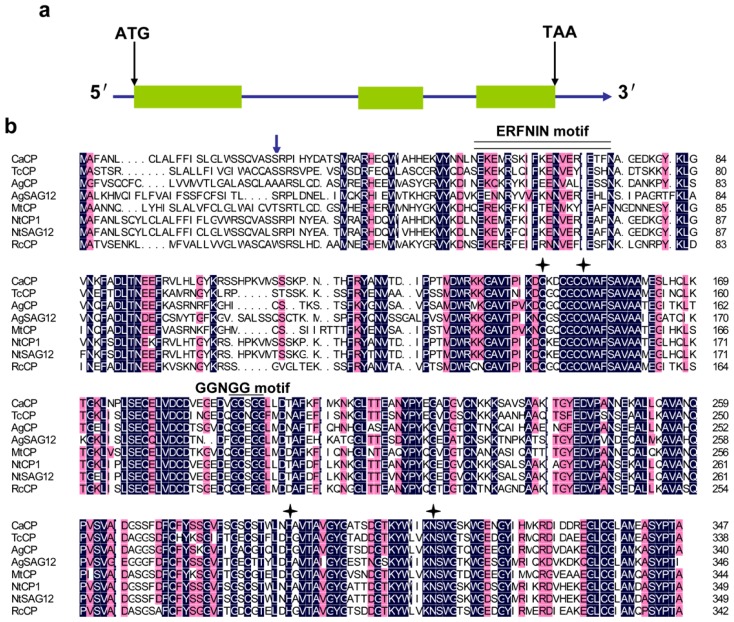
Gene structure of *CaCP* and multiple sequence alignment of the CaCP protein and other plant cysteine proteases (CPs). (**a**) Schematic representation the full-length DNA sequence of *CaCP*. The *CaCP* gene consists of two introns and three exons. Orang boxes represent exon sequences, blue lines between the exons indicate introns and both ends of the lines correspond to 5′- and 3′-UTR; (**b**) Multiple sequence alignment of CaCP and other plant CPs using DNAMAN software (Lynnon Biosoft, Vaudreuil, QC, Canada). Dark-blue shading and gray shading reflect 100% and 75% amino acid residues conservation, respectively. The arrow indicates the cleavage site of the signal peptide; the double line indicates the ERFNIN motif; the single line indicates the GCNGG motif, and the catalytic triad Cys, His, and Asn and also the Glu active site residue are indicated by the asterisks. Besides CaCP, other amino acid sequences included in this alignment were NtCP1 (ADV41672.1), NtSAG12 (AAW78661.2), TcCP (EOY28020.1), RcCP (XP_002523447.1), MtCP (XP_003612210.1), AgCP (AAA50755.1) and AtSAG12 (AAC49135.1). Ca, *Capsicum annuum* L; Nt, *Nicotiana tabacum*; Tc, *Theobroma cacao*; Rc, *Ricinus communis*; Dc, *Daucus carota*; Mt, *Medicago truncatula*; Ag, *Alnus glutinosa*; At, *Arabidopsis thaliana*.

A sequence of 662 bp was obtained from GenBank, which has high similarity to *Nicotiana tabacum senescence-specific CP* gene (designated *NtCP1*, accession number: AY881011). Subsequently, a 857 bp 5′-end fragment was amplified by 5′ RACE and a 420 bp 3′-end cDNA sequence was cloned by 3′ RACE. Finally, a full-length cDNA designated *CaCP* was assembled using the Contig Express software (Invitrogen, Carlsbad, CA, USA). The transcript consists of 1316 nucleotides, including a 5′-untranslated region (UTR) of 67 bp, an open reading frame (ORF) of 1044 bp and a 3′-UTR of 205 bp (GenBank accession number: KC176710). *CaCP* was predicted to encode a 347 amino acid protein with a theoretical molecular weight (MW) of 38.4 kDa and calculated isoelectric point (pI) of 6.56. The full-length DNA sequence of *CaCP* was 2143 bp. Analysis revealed that two introns were found at position 520–1132 and 1399–1534, respectively (Figure 1a, [32]).

The CBS prediction server’s program analysis indicated that CaCP contained a signal peptide region and a transmembrane helix at the *N* terminus (position M1–S25 and L17–T33, respectively). Structure analysis revealed that CaCP belongs to papain-like CPs superfamily, containing a highly conserved interspersed ERFNIN motif (position E55–N74). Additionally, a GCNGG motif (GCSGG in *Capsicum annuum*, G192–G196) was identified in CaCP. Except for the central Asn (N194) residue, this motif was invariant in all CPs reported. CaCP protein contained a conserved CP catalytic triad Cys (C153), His (H290) and Asn (N311), as well as the conserved Glu residue (Q147) (Figure 1b).

Alignment of CaCP protein sequence with already-reported CP amino acid sequences in the NCBI (Bethesda, MD, USA) database showed high homology to the group of papain-like CPs (Figure 1b). These included NtCP1 (similarity 87%, accession number: ADV41672.1), NtSAG12 (86%, AAW78661.2), TcCP (65%, EOY28020.1), RcCP (64%, XP_002523447.1) and MtCP (61%, XP_003612210.1). However, *Ca*CP was less related to AgCP (59%, AAA50755.1) and AtSAG12 (54%, AAC49135.1).

**Figure 2.**
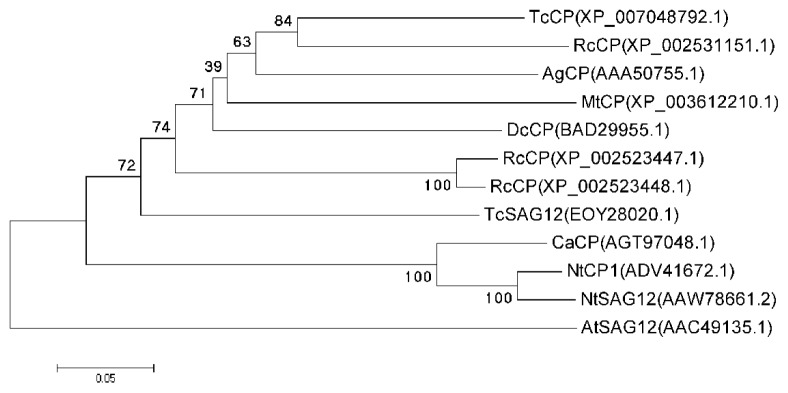
Phylogenetic analysis of CaCP protein and CP proteins of other plant species. The phylogenetic tree was constructed by the neighbor-joining method using MEGA5.05 software (The Biodesign Institute). Branches were labelled with the protein names and GenBank accession numbers. The tree was displayed as a phylogram in which branch lengths are proportional to distance. Numbers on the branches represent bootstrap values (for 1000 replicates). Ca, *Capsicum annuum* L; Nt, *Nicotiana tabacum*; Tc, *Theobroma cacao*; Rc, *Ricinus communis*; Dc, *Daucus carota*; Mt, *Medicago truncatula*; Ag, *Alnus glutinosa*; At, *Arabidopsis thaliana*.

A phylogenetic tree, constructed using the MEGA5.05 software (The Biodesign Institute, Tempe, AZ, USA), was used to investigate the evolutionary relatedness of the CaCP amino acid sequence to CP proteins of other plants (Figure 2). It showed CaCP protein was located in a clade in which CaCP, NtCP1 and NtSAG12 were tightly clustered. Other homologous proteins, including TcSAG12, RcCP1, RcCP2, DcCP, MtCP AgCP, among others, were clustered into another large cluster. The close relationship between CaCP, NtCP1 and NtSAG12 suggested that they may have a similar function.

### 2.2. Subcellular Localization of CaCP Protein

**Figure 3.**
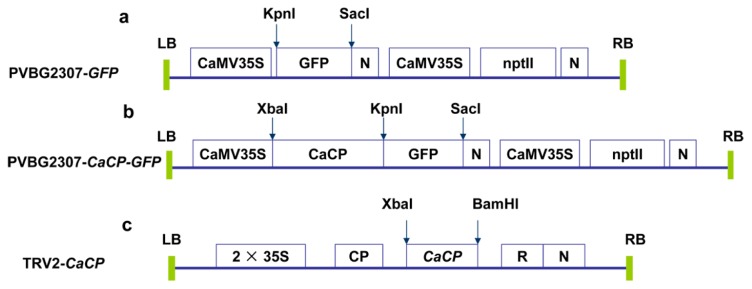
Schematic representation of the vector PVBG2307-*GFP* and PVBG2307-*CaCP-GFP* used for CaCP subcellular localization (**a**,**b**); and vector TRV2-*CaCP* used for *CaCP* gene silencing (**c**). LB: left borders of the T-DNA; RB: right borders of the T-DNA; CaMV35S: CaMV35S promoter; *CaCP: Capsicum annuum* cysteine proteinase; GFP: green fluorescent protein; nptII: neomycin phosphotransferase; N: nos-terminator; 2 × 35S: two copies of the cauliflower mosaic virus 35S promoter; CP: coat protein; R: ribozyme.

**Figure 4.**
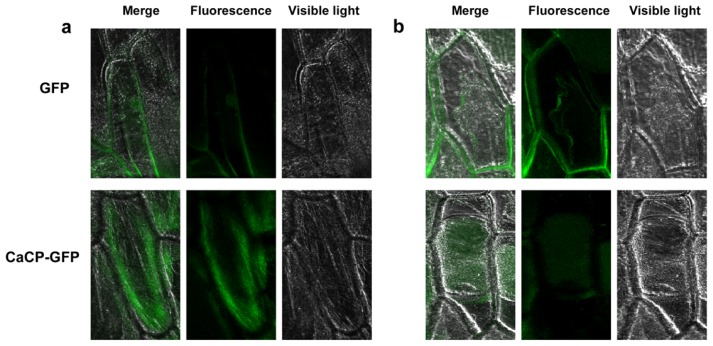
Subcellular localization of the GFP protein (**upper**) and CaCP-GFP fusion protein (**lower**) in onion epidermal cells. (**a**) The fluorescence images of non-plasmolysed cells; (**b**) The fluorescence images of plasmolysed cells.

To elucidate the subcellular localization of CaCP protein, *Agrobacterium tumefaciens* strain GV3101 carrying PVBG2307-*CaCP-GFP* or PVBG2307-*GFP* (control) construct was inoculated into onion epidermal cell (Figure 3a,b). After 36–48 h of incubation of transformed samples, the control cell transformed with GFP exhibited fluorescence in the entire region of the cell. The fluorescence of CaCP–GFP fusion protein was detected within the plasma membrane of the epidermal cell, where it had a large central vacuole (Figure 4a). Previous studies have reported that many senescence-associated CPs was localized in the vacuole [25,33–35]. To further identify whether CaCP was localized in the central vacuole of the onion epidermal cell, plasmolysis occurred in the epidermal cell which transformed with CaCP–GFP. The *Ca*CP–GFP fusion protein fluoresced in the central vacuole of the plasmolysed cell (Figure 4b). These results suggested that CaCP was localized in the central vacuole.

### 2.3. Expression of the CaCP Gene in Different Pepper Tissues and during Leaf Development

**Figure 5.**
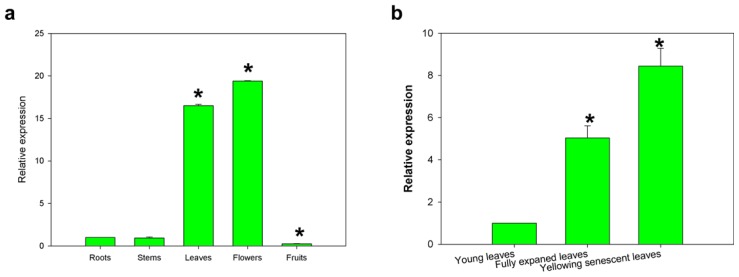
(**a**) Expression pattern of *CaCP* in different pepper tissues. Samples were collected from roots, stems, green leaves, flowers and mature fruits. Relative expression levels of the *CaCP* transcripts were determined in different tissues in comparison to that in roots; (**b**) The expression profiles of *CaCP* during pepper leaf developmental stage. RNA was extracted from young leaves, fully expanded leaves and senescent leaves, respectively. Error bars represent SD for three biological replicates. Asterisks indicate a significant difference (*p* < 0.05) from the roots.

To determine the expression profiles of the *CaCP* gene in different tissues, total RNA was extracted from the roots, stems, leaves, flowers and mature fruits (Figure 5a). qRT-PCR analyses revealed *CaCP* transcripts were detectable in all tissues and its abundance in the leaves and flowers was 16.5- and 19.3-fold higher, respectively, than that in the roots. However, *CaCP* expression level in the stems and mature fruits was lower than in the roots. For different leaf developmental stages, leaves were harvested at the young, fully expanded mature green and yellowing senescent stage, respectively. *CaCP* transcripts were detectable in young leaves and increased following the growth of leaf age, reaching a maximum in senescent leaves (Figure 5b).

### 2.4. Induction of the CaCP Gene in Pepper Leaves by Signaling Molecules, Abiotic and Biotic Stresses

**Figure 6.**
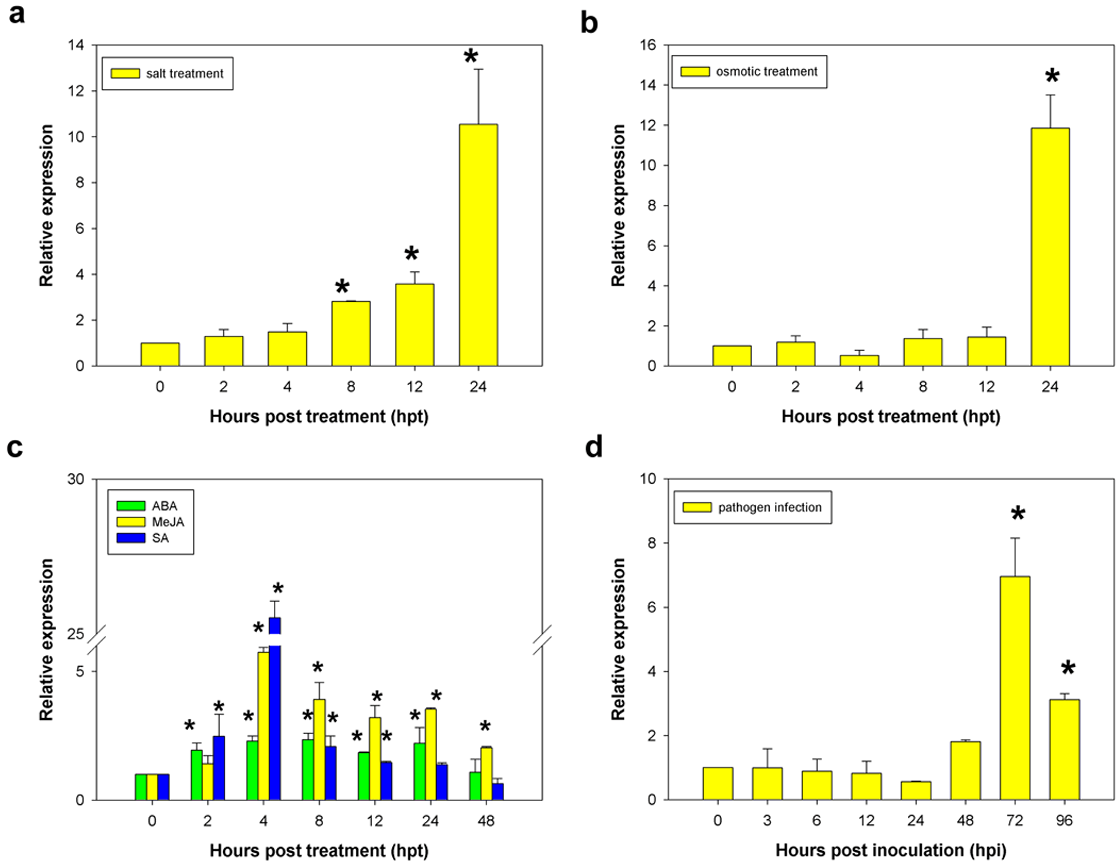
Expression profiles of *CaCP* in response to biotic and abiotic stresses including salt stress (**a**); osmotic stress (**b**); exogenous phytohormones (**c**) and pathogen infection (**d**). The relative transcriptional expression of *CaCP* was calculated in various treated leaves in comparison to that in the mock controls (0 h). Error bars represent SD for three biological replicates. Asterisks indicate a significant difference (*p* < 0.05) from 0 h p.t. or 0 h p.i.

In order to examine the expression pattern of *CaCP* in response to signaling molecules, in addition to abiotic and biotic stresses, B12 cultivar plants at the six-leaf stage were treated with phytohormones and various stresses, including abscisic acid (ABA), methyl jasmonate (MeJA), salicylic acid (SA), sodium chloride (NaCl), mannitol, and *Phytophthora capsici* infection, and analyzed by qRT-PCR. The *CaCP* expression level began to increase gradually at 2 h and upregulated 3.6-fold at 12 h after NaCl treatment, compared to the control (0 h). *CaCP* transcripts were induced quickly after 12 h and reached a maximum of 10.5-fold higher than that of the control at 24 h (Figure 6a). With mannitol treatment, the *CaCP* transcripts remained fairly static from 2 to 12 h except for a slight downregulation at 4 h. Then the transcripts increased rapidly and peaked (11.8-fold) at 24 h (Figure 6b).

Previous studies have shown that phytohormones such as ABA, MeJA, and SA were involved in response to various abiotic and biotic stresses and promoted leaf senescence in many plants [2,36–40]. To examine whether these three phytohormones can induce *CaCP* expression, pepper leaves were treated with ABA, MeJA, and SA, respectively. As shown in Figure 6c, *CaCP* transcription was increased gradually at 2 h and peaked by 8 h at a 2.3-fold greater level than the control (0 h), then declined slowly and finally returned to a normal level at 48 h following ABA treatment. Interestingly, there was a slight upregulation at 24 h. Compared with ABA treatment, MeJA and SA treatment could cause more significant changes in the expression level of *CaCP* in different degrees. MeJA could induce quickly the expression of the *CaCP* gene within the first 4 h after treatment. Compared to the control, *CaCP* transcripts were detected at the highest level (5.7-fold) at 4 h and then decreased gradually after 8 h. The treatment of SA induced rapidly the *CaCP* transcript abundance at 4 h to 25.5-fold higher than the control, but it declined sharply to 2.1-fold higher at 8 h. This expression level then decreased slowly from 8 to 48 h.

In pepper leaves challenged by the virulent *Phytophthora capsici* HX-9 stain, the *CaCP* transcriptional level was slow gradually declined from 3 to 24 h, compared with the control (Figure 6d). The transcripts then upregulated rapidly and peaked to 6.9-fold at 72 h. Subsequently, the *CaCP* expression level was decreased to 3.1-fold at 96 h, which was the lowest actual time point comparing to the control.

### 2.5. Phenotype and Silencing Efficiency of CaCP-Silenced Plants

**Figure 7.**
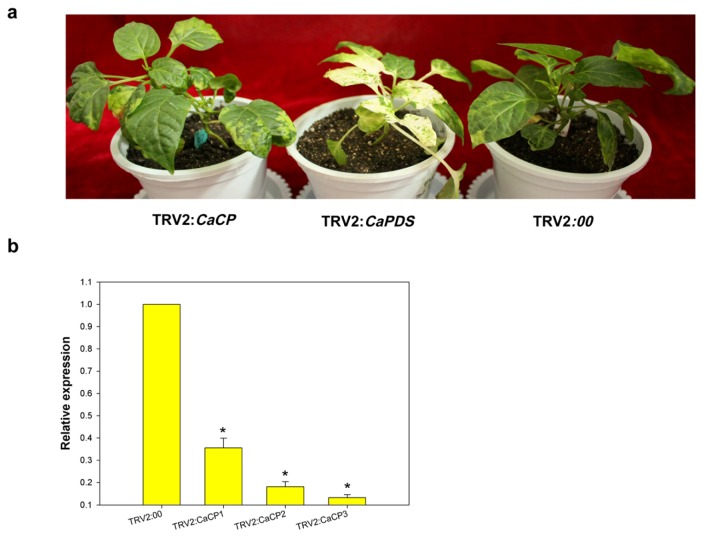
Efficiency of *CaCP* in pepper plants using a TRV-based VIGS system. (**a**) Phenotypes of gene-silenced pepper plants 45 days after inoculation. **Left**: *CaCP*-silenced plant (TRV2:*CaCP*); **middle**: *CaPDS*-silenced plant (TRV2:*CaPDS*); **right**: control plant (TRV2:00); (**b**) Quantitative real time-PCR analysis of *CaCP* expression levels in leaves of *CaCP*-silenced plants (TRV2:*CaCP1*, TRV2:*CaCP2* and TRV2:*CaCP3* indicates three biological levels of efficiency of *CaCP* gene silencing) and control plants (TRV2:00) 45 days after inoculation. Error bars represent SD for three biological replicates. Asterisks indicate a significant difference (*p* < 0.05) compared to TRV2:00.

The aforementioned results suggest that *CaCP* is involved in the response to various abiotic and biotic stresses in pepper plant. In order to get more details of the function of *CaCP* in pepper, a tobacco rattle virus (TRV)-based VIGS technique was used to silence the *CaCP* gene (Figure 3c). An empty vector was applied to plants (TRV2:00) as a negative control. The *CaPDS*-silenced plants (TRV2:*CaPDS*), in which the endogenous *phytoene desaturase* (*PDS*) gene was silenced to cause photobleaching, were used as positive controls. Mild viral symptom began to appear in all plants at three weeks after the *Agrobacterium* inoculation, and the *CaPDS*-silenced plants exhibited the photobleached phenotype. These results suggested that the TRV-induced VIGS system was successfully applied in this experiment. Simultaneously, as shown in Figure 7a, no morphological distinction was observed between the *CaCP*-silenced plants (TRV2:*CaCP*) and the empty vector control plants (TRV2:00) 45 days after inoculation.

To clarify the efficiency of *CaCP* gene silencing by VIGS, qRT-PCR was carried out on RNA extracted from the leaves of *CaCP*-silenced plants (TRV2:*CaCP*) and empty vector control plants (TRV2:00). As shown in Figure 7b, the abundance of *CaCP* transcripts were significantly reduced to different extents in *CaCP*-silenced plants compared to the empty vector control. The results indicated that the *CaCP* gene was partially silenced.

### 2.6. Enhanced Tolerance of CaCP-Silenced Pepper Plants to Salt Stress

**Figure 8.**
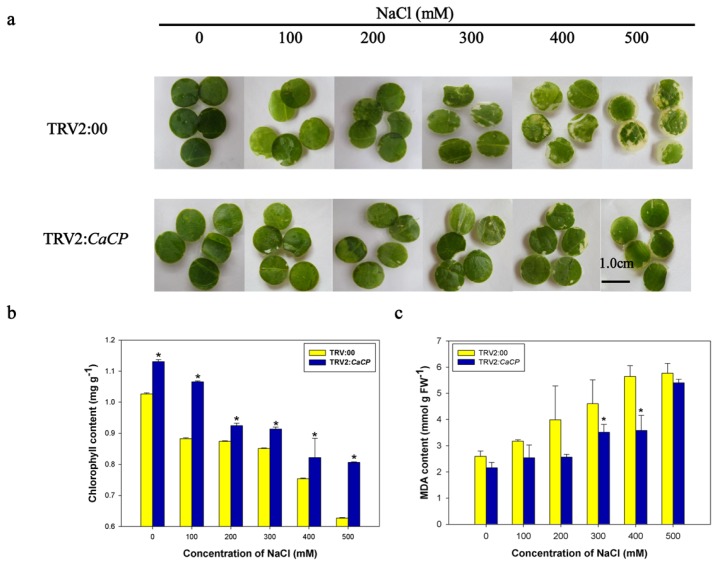
Enhance tolerance of *CaCP*-silenced pepper plants to salt stress. Phenotypes (**a**); chlorophyll contents (**b**) and MDA contents (**c**) of leaf discs of the gene-silenced plants in response to high salt stress. Leaf discs from the gene-silenced pepper leaves were floated in different concentrations of NaCl solutions (0, 100, 200, 300, 400 and 500 mM) for 3 days at 26 °C with continuous fluorescent lighting. The scale bar represents 1.0 cm. Error bars represent SD for three biological replicates. Asterisks indicate a significant difference (*p* < 0.05) compared to TRV2:00 leaves.

Senescence of leaf was manifested in chlorophyll breakdown and reactive oxygen species (ROS) accumulation [40,41]. ROS-generated lipid peroxidation (reflected by malondialdehyde (MDA) content) is an inherent feature of senescing cells and a source of ROS [42]. To determine whether the *CaCP* led to reduced tolerance to salt stress, the leaf discs from empty vector control-treated (TRV2:00) and *CaCP*-silenced (TRV2:*CaCP*) plants were exposed to various concentrations (0, 100, 200, 300, 400 and 500 mM) of NaCl solution with continuous lighting for 3 days. The chlorophyll and MDA content were measured. The leaf discs of the empty vector control plants were more severely bleached after 3 days of salt treatment, compared to those of *CaCP*-silenced plants (Figure 8a). Consistently, the total chlorophyll content in the *CaCP*-silenced leaves was higher than in the empty vector control plants (Figure 8b). The MDA content was gradually elevated with NaCl concentration increase, and its increase rate in the control plants was higher than that in *CaCP*-silenced plants (Figure 8c). These results demonstrated that *CaCP* was a negative regulator in pepper resistance to salt stress.

### 2.7. Silencing of CaCP Enhanced Tolerance to Osmotic Stress in Pepper Plants

**Figure 9.**
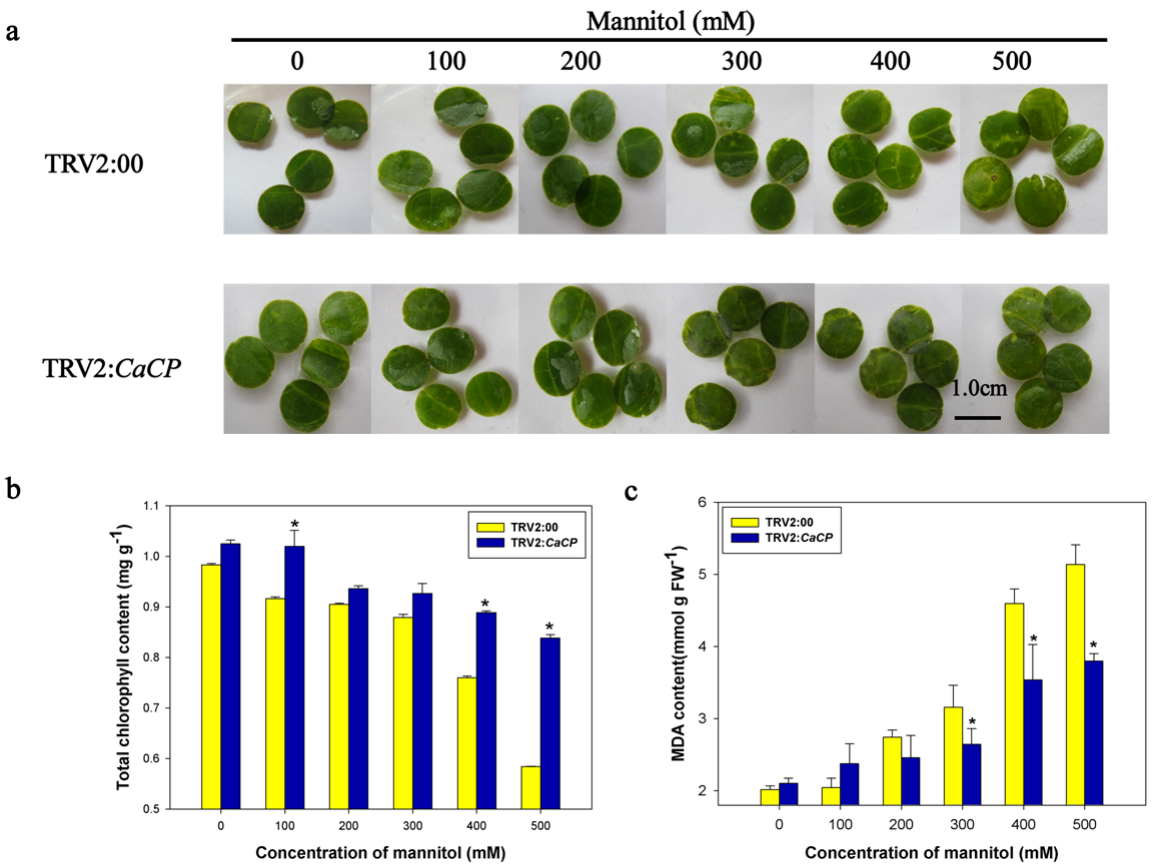
Enhanced tolerance of *CaCP*-silenced pepper plants to osmotic stress. Phenotypes (**a**); chlorophyll contents (**b**) and MDA contents (**c**) of leaf discs of the gene-silenced plants in response to high osmotic stress. Leaf discs from the gene-silenced pepper leaves were floated in different concentrations of mannitol solutions (0, 100, 200, 300, 400, and 500 mM) for 3 days at 26 °C with continuous fluorescent lighting. The scale bar represents 1.0 cm. Error bars represent SD for three biological replicates. Asterisks indicate a significant difference (*p* < 0.05) compared to TRV2:00 leaves.

To analyze whether the silencing of *CaCP* in pepper plants enhanced osmotic stress tolerance, mannitol was used in the leaf discs of gene-silenced plants. As shown in Figure 9a, the color of the leaf discs of the empty vector control plants turned yellow and a few leaf discs blades appeared to be a bleached phenotype under treatment with higher mannitol concentrations (400 and 500 mM), whereas the color of *CaCP*-silenced leaves remained green under all treatment conditions. Furthermore, chlorophyll breakdown in the empty vector control leaves was observed to occur faster than that in *CaCP*-silenced leaves 3 days after treatment with 400 or 500 mM mannitol (Figure 9b), and the MDA content of empty vector control leaves was also higher than that of *CaCP*-silenced leaves (Figure 9c). The findings indicated that silencing of *CaCP* was able to enhance tolerance pepper to high osmotic stresses.

### 2.8. Discussion

CPs are one of important proteolytic enzymes involving protein turnover, PCD, and degradation of stress-damaged proteins in higher plants [43,44]. In the present study, we isolated a CP gene from pepper plant, named *CaCP.* The predicted CaCP protein contained a signal peptide region that would be processed prior to enzyme activation. The CaCP protein also contained a conserved ERFNIN motif and a GCNGG motif that existed in CP homologs. Moreover, three highly conserved catalytic residues Cys (C153), His (H290) and Asn (N311) constitute the catalytic triad of CaCP protein, and another conserved Glu residue (Q147) is involved in maintaining an active enzyme conformation. Sequence alignment and phylogenetic analysis demonstrated that CaCP belonged to papain-like superfamily and shared high identity with known CP proteins from other plants. These results showed that CaCP should function as a CP.

The vacuole of plants contains most of the hydrolytic activity of the cell [45]. In cell suspension cultures of *Acer* the vacuole is involved in the recycling of cytosolic proteins and degradation of abnormally synthesized proteins [36,46]. A number of endogenous plant CPs associated with senescence have the relatively acidic pH optima, indicating they are localized in the vacuole *in vivo* [33,34]. Here, CaCP was localized to the vacuole of the cell, which is consistent with the vacuolar localization of senescence-associated SENU3 and SENU4 of tomato and four CPs of wheat [26,35]. The activities of four vacuolar CPs were increased during senescence under different conditions in wheat. These results suggested that vacuolar CPs may be involved in protein degradation during senescence.

Tissue-specific analysis showed that *CaCP* is expressed ubiquitously within all tested tissues, but *CaCP* gene exhibited a significantly higher expression level in leaves and flowers than in roots, stems and mature fruits. *CaCP* transcripts were detected in young leaves and continuously increased during leaf development. These results were similar to *SEN2* and *SEN3*, which were expressed in all tissues, but the highest transcripts were in leaves of tomato [26]. The *SEN2* and *SEN3* genes were expressed at low levels in the young, fully expanded leaves and upregulated during leaf senescence. The expression profile of *CaCP* is different with most homologous *CP* genes from other plants, which were involved in processing and degradation of seed storage proteins, fruit ripening and in legume nodule development [7,10,13]. *CsCP* transcripts were detectable in different organs and showed the highest level in peel pitting of navel orange fruit [10]. *PhCP10* was detected only in senescing tissues in petunia corollas [8]. Previous studies found that the major role of CPs in plant senescence is turnover and remobilization of cellular materials out of dying cells into other developing tissues [35,42]. These results suggested that *CaCP* was a senescence-associated gene, which was developmentally regulated and involved in turnover and remobilization of protein during leaf senescence in pepper plant.

Leaf senescence is not only governed by the developmental age but also modulated by phytohormones and environmental stresses. This is because the mechanisms of developmental senescence and those associated with defense responses to phytohormones and environmental stresses are thought to overlap [47]. Previous studies have shown that the change of *CP* gene expression is associated with environmental stresses in plants. For example, the senescence-associated gene *LsCP2* was expressed in senescent leaves and strongly induced in lettuce by a *Verticillium dahliae* attack [17]. Likewise, in the current study, the *CaCP* gene in pepper leaves was induced by phytohormones (ABA, MeJA or SA). The results indicated that *CaCP* may be involved in ABA-, JA- and SA-dependent signaling pathways. Results of Lim *et al.* [2] showed that the change of transcriptome mediated by the SA pathway was highly similar to that mediated by age-dependent senescence. SA may be involved in the regulation of drought-induced leaf senescence in perennials as SA accumulation preceded chlorophyll loss and nitrogen mobilization [48]. Here, salt and osmotic stresses as well as *Phytophthora capsici* infection strongly induced the *CaCP* gene expression in pepper leaves. It is likely that salt, mannitol and *Phytophthora capsici*-induced SA accumulation may trigger *CaCP* transcripts in pepper plants.

Leaf senescence, induced by age and various stresses, is involved in degradation of chlorophyll and other macromolecules such as proteins, lipids, *etc*. [3]. The process of protein degradation is initiated by ROS and involves the action of proteolytic enzymes such as CPs [42]. Dark-induced senescence in the leaves of apple was reflected in chlorophyll breakdown and an increase in the levels of ROS, with the induction of a senescence-associated *CP* gene [49]. In this research, chlorophyll content in the *CaCP*-silenced leaves decreased slower than in the empty vector control leaves 3 days after treatment with salt and high mannitol. In contrast, the level of lipid peroxidation in the empty vector control was increased faster than that in the *CaCP*-silenced leaves leaves. These results demonstrated that *CaCP* played a negative regulation role in pepper plant defense response to salt and osmotic stresses, thus delaying leaf senescence. The results of Prins *et al.* [36,50] have shown that chloroplast proteins, particularly Rubisco and Rubisco activase, are the major targets of leaf CPs. Overexpressing a rice cysteine proteinase inhibitor in tobacco could prevent Rubisco degradation and slower senescence, suggesting the importance of CPs in senescence.

## 3. Experimental Section

### 3.1. Plant Material and Growth Condition

The B12 pepper cultivar was employed in this study. This cultivar was selected by a pepper research group in the College of Horticulture, Northwest A&F University (Yangling, China). Pepper seeds were treated with warm water (55 °C) for 20 min and then incubated at 28 °C to accelerate germination under dark conditions until budding. The germinated seeds were sown in pots containing compost. Seedlings were grown in a chamber under temperature conditions at a 25/21 °C day/night temperature cycle, 60% relative humidity, and a photoperiod cycle of 16/8 h light/dark.

### 3.2. Plant Treatments

For qRT-PCR analyses, pepper plants at the six-leaf stage were treated with signaling molecules, abiotic and biotic stresses. For treatment with signaling molecules, leaves were sprayed with 0.57 mM ABA, 1 M MeJA or 5 mM SA. After 0, 2, 4, 8, 12, 24 and 48 h of treatment, pepper leaves were sampled. For salt and osmotic stresses, whole plants were uprooted from the soil, and their roots were soaked in 400 mM NaCl and 400 mM mannitol, respectively. After 0, 2, 4, 8, 12 and 24 h of treatment, leaves were collected. For the fungal pathogen treatment, pepper plants were inoculated with *Phytophthora capsici* (virulent strain HX-9) by the root-drenching method, the plants were incubated in a growth chamber at 28 °C under a photoperiod cycle of 16/8 h light/dark with 60% relative humidity [51]. Treated leaves were harvested at 0, 3, 6, 12, 24, 48, 72 and 96 h intervals. All samples were frozen immediately in liquid nitrogen and kept at −80 °C prior to RNA extraction.

To analyze whether the *CaCP* gene would respond to abiotic stresses, leaf discs (1.0 cm in diameter) were excised from the gene-silenced pepper leaves and were floated in different concentrations of NaCl or mannitol solutions (0, 100, 200, 300, 400 and 500 mM). The treatment was controlled at 26 °C with continuous fluorescent lighting for 3 days to test for salt and osmotic stresses of the gene-silenced plants.

### 3.3. Measurement of Chlorophyll Content and MDA Content

Total chlorophyll was extracted from the treated samples and calculated by a spectrophotometric method as described by Arkus *et al.* [52]. The MDA content of treated leaves was measured according to the method of Buege *et al.* [53]. All measurements were replicated three times.

### 3.4. Isolation of RNA and Genomic DNA

Total RNA was extracted from different pepper tissues without treatment and from leaves with different stress treatments using the Trizol (Invitrogen, Carlsbad, CA, USA) method. Contaminated genomic DNA was digested by RNase-free DNase I (Promega, Madison, WI, USA). Genomic DNA was extracted from pepper mature leaves using CTAB method. RNA and DNA concentrations were estimated by measuring the absorbance at 260 and 280 nm using a NanoDrop instrument (Thermo Scientific NanoDrop 2000C Technologies, Pittsburgh, PA, USA).

### 3.5. First-Strand cDNA Synthesis and qRT-PCR Analysis

The first-strand cDNA was synthesized using the PrimeScript™ Kit according to the manufacturer’s recommendation (TaKaRa, Tokyo, Japan). qRT-PCR was performed using SYBR^®^ Premix Ex Taq™ II (TaKaRa) with slight modifications as described by Wang *et al.* [54]. The 2^−ΔΔ^*^C^*^t^ comparative threshold method was used to calculate relative gene expression levels [55]. All reactions were performed in triplicate, and the *CaUBI3* gene (accession number: AY486137.1) was used as an internal control (reference gene) in this study (Table 1).

### 3.6. Cloning and Sequence Analysis of the CaCP Gene

A full-length cDNA of the putative *CaCP* gene was obtained by performing 5′ and 3′ rapid amplification of cDNA ends (RACE) using Smart RACE cDNA amplification kit (Clontech, Mountain View, CA, USA). Following the reported sequence (accession numbers: GD094760) of partial cDNA fragment, a set of gene-specific primers that were geared towards 5′ and 3′ RACE were designed (Table 1). A full-length cDNA sequence with a complete ORF was assembled using the Contig Express software and BLAST online software. A pair of primers (DNA-*CaCP*F and DNA-*CaCP*R), which was designed according to the complete cDNA sequence, was used to isolate the full-length DNA sequence of *CaCP*.

The pI and MW of CaCP protein were analyzed with the pI/MW program, and its secondary structure was predicted using the Scratch Protein Predictor online program. Prediction of the transmembrane, signal peptide and its cleavage site were carried out using the CBS prediction server’s online program. Multiple sequence alignments were performed with the DNAMAN software (Lynnon Biosoft), and a phylogenetic tree was constructed using the MEGA5.05 program (The Biodesign Institute) with the neighbor-joining method.

### 3.7. VIGS Assay of CaCP in Pepper Plant

The TRV-based VIGS system was used for *CaCP* gene silencing in pepper plants as previously described by Liu *et al.* [30]. The cDNA fragment derived from the coding sequence and the 3′-UTR (323 bp) were cloned into pTRV2 vector construct the recombinant plasmid TRV2-*CaCP*. PDS is an enzyme involved in the carotenoid biosynthesis pathway, and silencing of the *PDS* gene causes photobleaching. The *CaPDS* gene (*phytoene desaturase* from *C. annuum*, accession number: X68058.1) was used in this study, and the TRV2-*CaPDS* vector was designed by our laboratory. TRV2-*CaPDS* and pTRV2 vectors were used as controls for the TRV infection. *Agrobacterium tumefaciens* strain GV3101 harboring pTRV1 was respectively mixed with pTRV2, TRV2-*CaPDS* or TRV2-*CaCP* at a 1:1 ratio, then, the mixtures were inoculated into the fully expanded cotyledons of the B12 pepper cultivars. Plants were placed in a growth chamber at a constant temperature of 18 °C and 60% relative humidity for 2 days. Subsequently, the plants were grown at 23 °C under a 16/8 h light/dark photoperiod cycle and 60% relative humidity. At 45 days after inoculation, the leaves were used for abiotic stresses treatment.

### 3.8. Subcellular Localization of the CaCP Protein

The full-length *CaCP* coding region was cloned into PVBG2307 vector under the control of CaMV 35S promoter and fused in the 3′ region with the green fluorescence protein (GFP) gene to yield PVBG2307-*CaCP-GFP* (Table 1). The PVBG2307-*CaCP-GFP* and PVBG2307-*GFP* (control) plasmids were transient expressed in onion epidermal cell using *Agrobacterium tumefaciens-*mediated transformation with slight modifications as described by Li *et al.* [56]. All samples were incubated for 36–48 h at 28 °C in darkness. Confocal laser scanning microscopy was performed for the subcellular localization of the CaCP protein. Onion epidermal cells were plasmolysed in 0.8 M mannitol for 15 min [57].

### 3.9. Primers Used in this Study

Primer sequences used for cloning, qRT-PCR analysis, subcellular localization and VIGS in this report are listed in Table 1.

### 3.10. Statistical Analysis

All data are presented as the mean ± standard deviation (SD) of three replicates and were analyzed using Statistical Analysis System software (SAS Institute, version 8.2, Cary, NC, USA).

## 4. Conclusions

In summary, a *CP* gene was identified from pepper. An expression profile of *CaCP* in different tissues and during leaf development revealed that *CaCP* was associated with leaf senescence. *CaCP* expression pattern during various stress treatments implicated its involvement in abiotic and biotic stresses. Silencing of *CaCP* in pepper led to enhanced resistance to salt and osmotic stresses. Taken together, these data suggest that *CaCP* plays an important role during leaf development, as well as in salt- and osmotic-induced leaf senescence. In future research, *CaCP-*overexpressing transgenic plant can be used to study in more detail the function of *CaCP* in pepper.

## Figures and Tables

**Table 1.** Primer sequences in this study.

Primers	Sequence(5′–3′)
*Cloning of CaCP cDNA sequence*	

EST-*CaCP*F	TACAGGATATGAAGATGTCCCAGC
EST-*CaCP*R	AAGCCTCCATGGCAAGTCC
5′RACE-*CaCP*GSP	GTTTTCACCCCATTTACTGCCCC
5′RACE-*CaCP*NGSP	CCACTGACACTGGTTGATTTGCC
3′RACE-*CaCP*GSP	GATATGAAGATGTCCCAGCCAAC
3′RACE-*CaCP*NGSP	GTGGTGTATTCAGTGGATCATGC

*Cloning of CaCP DNA sequence*	

DNA-*CaCP*F	TAGTTGTTCTATAATGGCCTTTGCA
DNA-*CaCP*R	ACAATTTAGAAGCTGGCACCATT

*quantitative real-time PCR*	

RT-*CaCP*F	TTGTTCTATAATGGCCTTTGCA
RT-*CaCP*R	TCATAGTGAATTGGACGTGGTG
*CaUBI3*F	TGTCCATCTGCTCTCTGTTG
*CaUBI3R*	CACCCCAAGCACAATAAGAC

*Virus-induced gene-silencing (VIGS) vector construction*	

TRV-*CaCP*F	GAGTAGCTTCGATTTCCAGTTC
TRV-*CaCP*R	CAAATCCACAACGTATTCACAT
*CaPDS*F	TGTTGTCAAAACTCCAAGGTCTGTA
*CaPDS*R	TTTCTCCCACTTGGTTCACTCTTGT

*PVBG2307-CaCP-GFP vector construction*	

GFP-*CaCP*F	TTCTATAATGGCCTTTGCAAACC
GFP-*CaCP*R	GGCAGTGGGATAAGAAGCCTC

## References

[1] Smart C. (1994). Gene expression during leaf senescence. New Phytol.

[2] Lim P.O., Kim H.J., Nam H.G. (2007). Leaf Senescence. Annu. Rev. Plant Biol.

[3] Guo Y., Cai Z., Gan S. (2004). Transcriptome of Arabidopsis leaf senescence. Plant Cell Environ.

[4] Makino A., Osmond B. (1991). Effects of nitrogen nutrition on nitrogen partitioning between chloroplasts and mitochondria in pea and wheat. Plant Physiol.

[5] Staswick P.E. (1994). Storage proteins of vegetative plant tissues. Annu. Rev. Plant Phys.

[6] Robertsa I.N., Caputo C., Criado M.V., Funk C. (2012). Senescence-associated proteases in plants. Physiol. Plantarum.

[7] Toyooka K., Okamoto T., Minamikawa T. (2000). Mass transport of a proform of a KDEL-tailed cysteine proteinase (SH-EP) to protein storage vacuoles by endoplasmic reticulum-derived vesicle is involved in protein mobilization in germinating seeds. J. Cell Biol.

[8] Jones M.L., Chaffin1 G.S., Eason J.R., Clark D.G. (2005). Ethylene-sensitivity regulates proteolytic activity and cysteine protease gene expression in petunia corollas. J. Exp. Bot.

[9] Watanabe Y., Matsushima S., Yamaguchi A., Shioi Y. (2009). Characterization and cloning of cysteine protease that is induced in green leaves of barley. Plant Sci.

[10] Fan J., Yang Y.W., Gao X., Deng W., Falara V., Kanellis A.K., Li Z.G. (2009). Expression of a senescence-associated cysteine protease gene related to peel pitting of navel orange (*Citrus sinensis* L. Osbeck). Plant Cell Tissue Org.

[11] Zhang X.M., Wang Y., Lv X.M., Li H., Sun P., Lu H., Li F.L. (2009). NtCP56, a new cysteine protease in *Nicotiana tabacum* L., involved in pollen grain development. J. Exp. Bot.

[12] Naito Y., Fujie M., Usami S., Murooka Y., Yamada T. (2000). The involvement of cysteine proteinase in the nodule development in Chinese milk vetch infected with *Mesorhizobium huakuii* subsp. Rengei. Plant Physiol.

[13] Li Y.X., Zhou L., Li Y.G., Chen D.S., Tan X.J., Lei L., Zhou J.C. (2008). A nodule-specific plant cysteine proteinase, AsNODF32, is involved in nodule senescence and nitrogen fixation activity of the green manure legume *Astragalus sinicus*. New Phytol.

[14] Forsthoefel N.R., Cushman M.A.F., Ostrem J.A., Cushman J.C. (1998). Induction of a cysteine protease cDNA from *Mesembryanthemum crystallinum* leaves by environmental stress and plant growth regulators. Plant Sci.

[15] Simova S.L., Vaseva I., Grigorova B., Demirevska K., Feller U. (2010). Proteolytic activity and cysteine protease expression in wheat leaves under severe soil drought and recovery. Plant Physiol. Biochem.

[16] Maciel F.M., Salles C.M.C., Retamal C.A., Gomes V.M., Machado O.L.T. (2011). Identification and partial characterization of two cysteine proteases from castor bean leaves (*Ricinus communis* L.) activated by wounding and methyl jasmonate stress. Acta Physiol Plant.

[17] Klostermana S.J., Anchieta A., Garcia-Pedrajas M.D., Maruthachalam K., Hayes R.J., Subbarao K.V. (2011). SSH reveals a linkage between a senescence-associated protease and Verticillium wilt symptom development in lettuce (*Lactuca sativa*). Physiol. Mol. Plant Pathol.

[18] Shin R., Lee G.J., Park C.J., Kim T.Y., You J.S., Nam Y.W., Peak K.H. (2001). Isolation of pepper mRNAs differentially expressed during the hypersensitive response to tobacco mosaic virus and characterization of a proteinase inhibitor gene. Plant Sci.

[19] Karrer K.M., Peiffer S.L., DiTomas M.E. (1993). Two distinct gene subfamilies of cysteine proteinase gene. Proc. Natl. Acad. Sci. USA.

[20] Okamoto T., Shimada T., Hara-Nishimura I., Nishimura M., Minamikawa T. (2003). *C*-terminal KDEL sequence of a KDEL tailed cysteine proteinase (sulfhydryl-endopeptidase) is involved in formation of KDEL vesicle and in efficient vacuolar transport of sulfhydryl-endopeptidase. Plant Physiol.

[21] Ling J., Kojima T., Shiraiwa M., Takahara H. (2003). Cloning of two cysteine proteinases genes: CysP1 and CysP2, from soybean cotyledons by cDNA representational difference analysis. Biochim. Biophys. Acta.

[22] Granell A., Cercos M., Carbonell J., Barrett A.J., Rawlings N.D., Woessner J.F. (1998). Plant cysteine proteinases in germination and senescence. Handbook of Proteolytic Enzymes.

[23] Richau K.H., Kaschani F., Verdoes M., Pansuriya T.C., Niessen S., Stüber K., Colby T., Overkleeft H.S., Bogyo M., van der Hoorn R.A. (2012). Subclassification and biochemical analysis of plant papain-like cysteine proteases displays subfamily-specific characteristics. Plant Physiol.

[24] Guerrero C., Calle M., Reid M.S., Valpuesta V. (1998). Analysis of the expression of two thiolprotease genes from daylily (Hemerocallis spp.) during flower senescence. Plant Mol. Biol.

[25] Beyene G., Foyer C.H., Kunert K.J. (2006). Two new cysteine proteinases with specific expression patterns in mature and senescent tobacco (*Nicotiana tabacum* L.) leaves. J. Exp. Bot.

[26] Drake R., John I., Farrell A., Cooper W., Schuch W., Grierson D. (1996). Isolation and analysis of cDNAs encoding tomato cysteine proteases expressed during leaf senescence. Plant Mol. Biol.

[27] Lohman K.N., Gan S., John M.C., Amasino R.M. (1994). Molecular analysis of natural leaf senescence in *Arabidopsis thaliana*. Physiol. Plantarum.

[28] Ueda T., Seo S., Ohashi Y., Hashimoto J. (2000). Circadian and senescence-enhanced expression of a toabcco cysteine protease gene. Plant Mol. Biol.

[29] Chen G.H., Huang L.T., Yap M.N., Lee R.H., Huang Y.J., Cheng M.C., Chen S.C.G. (2002). Molecular characterization of a senescence-associated gene encoding cysteine proteinase and its gene expression during leaf senescence in sweet potato. Plant Cell Physiol.

[30] Liu Y., Schiff M., Dinesh-Kumar S.P. (2002). Virus-induced gene silencing in tomato. Plant J.

[31] Brigneti G., Martin-Hernandez A., Jin H., Chen J., Baulcombe D.C., Baker B., Jones J.D. (2004). Virus-induced gene silencing in *Solanum* species. Plant J.

[32] CM334 Genome Assembly, Pseudomolecules, Annotations and *C. chinense* Genome Assembly.

[33] Callis J. (1995). Regulation of protein degradation. Plant Cell.

[34] Srivalli B., Bharti S., Khanna-Chopra R. (2001). Vacuole cysteine proteases and ribulose-1,5-bisphosphate carboxylase/oxygenase degradation during monocarpic senescence in cowpea leaves. Photosynthetica.

[35] Martínez D.E., Bartoli C.G., Grbic V., Guiamet J.J. (2007). Vacuolar cysteine proteases of wheat (*Triticum aestivum* L.) are common to leaf senescence induced by different factors. J. Exp. Bot.

[36] Prins A., van Heerden P.D.R., Olmos E., Kunert K.J., Foyer C.H. (2008). Cysteine proteinases regulate chloroplast protein content and composition in tobacco leaves: A model for dynamic interactions with ribulose-1,5-bisphosphate carboxylase/oxygenase (Rubisco)vesicular bodies. J. Exp. Bot.

[37] Kong Z., Li M., Yang W., Xu W., Xue Y. (2006). A novel nuclear-localized CCCH-type zinc finger protein, OsDOS, is involved in delaying leaf senescence in rice (*Oryza sativa* L.). Plant Physiol.

[38] Morris K., Mackerness S.A., Page T., John C.F., Murphy A.M., Carr J.P., Buchanan-Wollaston V. (2000). Salicylic acid has a role in regulating gene expression during senescence. Plant J.

[39] Gepstein S., Thimann K.V. (1980). Changes in the abscisic acid content of oat leaves during senescence. Proc. Natl. Acad. Sci. USA.

[40] Rosenvasser S., Mayak S., Friedman H. (2006). Increase in reactive oxygen species (ROS) and in senescence-associated gene transcript (SAG) levels during dark-induced senescence of *Pelargonium* cuttings, and the effect of gibberellic acid. Plant Sci.

[41] Vanacker H., Sandalio L.M., Jimenez A., Palma J.M., Corpas F.J., Meseguer V., Gómez M., Sevilla F., Leterrier M., Foyer C.H. (2006). Role of redox regulation in leaf senescence of pea plants grown in different sources of nitrogen nutrition. J. Exp. Bot.

[42] Chopra K.R. (2012). Leaf senescence and abiotic stresses share reactive oxygen species-mediated chloroplast degradation. Protoplasma.

[43] Solomon M., Belenghi B., Delledonne M., Menachem E., Levine A. (1999). The involvement of cysteine proteases and protease inhibitor genes in the regulation of programmed cell death in plants. Plant Cell.

[44] Palma M.J., Sandalio M.L., Javier C.F. (2002). Plant proteases, protein degradation, and oxidative stress: role of peroxisomes. Plant Physiol. Biochem.

[45] De D.N. (2000). Functions of vacuoles. Plant Cell Vacuoles: An Introduction.

[46] Canut H., Alibert G., Carrasco A., Boudet A.M. (1986). Rapid degradation of abnormal proteins in vacuoles from *Acer pseudoplatanus* L. cells. Plant Physiol.

[47] Zhang X., Zhang Z., Li J., Wu L., Guo J., Ouyang L., Xia Y., Huang X., Pang X. (2011). Correlation of leaf senescence and gene expression/activities of chlorophyll degradation enzymes in harvested Chinese flowering cabbage (*Brassica rapa* var. parachinensis). J. Plant Physiol.

[48] Abreu M.E., Munné-Bosch S. (2008). Salicylic acid may be involved in the regulation of drought-induced leaf senescence in perennials: A case study in field-grown *Salvia officinalis* L. plants. Environ. Exp. Bot.

[49] Wang P., Yin L.H., Liang D., Li C., Ma F.W., Yue Z.Y. (2012). Delayed senescence of apple leaves by exogenous melatonin treatment: Toward regulating the ascorbate–glutathione cycle. J. Pineal Res.

[50] Otegui M.S., Noh Y.S., Martínez D.E., Petroff M.G.V., Staehelin L.A., Amasino R.M., Guiamet J.J. (2005). Senescence-associated vacuoles with intense proteolytic activity develop in leaves of *Arabidopsis* and soybean. Plant J.

[51] Wang J.E., Li D.W., Zhang Y.L., Zhao Q., He Y.M., Gong Z.H. (2013). Defence responses of pepper (*Capsicum annuum* L.) infected with incompatible and compatible strains of *Phytophthora capsici*. Eur. J. Plant Pathol.

[52] Arkus K.A.J., Cahoon E.B., Jez J.M. (2005). Mechanistic analysis of wheat chlorophyllase. Arch. Biochem. Biophys.

[53] Buege J.A., Aust S.D. (1978). Microsomal lipid peroxidation. Method Enzymol.

[54] Wang J.E., Liu K.K., Li D.W., Zhang Y.L., Zhao Q., He Y.M., Gong Z.H. (2013). A novel peroxidase *CanPOD* gene of pepper is involved in defense responses to *Phytophtora capsici* infection as well as Aabiotic stress tolerance. Int. J. Mol. Sci.

[55] Livak K.J., Schmittgen T.D. (2001). Analysis of relative gene expression data using real-time quantitative PCR and the 2^−ΔΔ^*^C^*^t^ Method. Methods.

[56] Li Z., Wang S., Tao Q.Y., Pan J.S., Si L.T., Gong Z.H., Cai R. (2012). A putative positive feedback regulation mechanism in *CsACS2* expression suggests a modified model for sex determination in cucumber (*Cucumis sativus* L.). J. Exp. Bot.

[57] Genovesi V., Fornalé S., Fry S.C., Ruel K., Ferrer P., Encina A., Sonbol F.M., Bosch J., Puigdomènech P., Rigau J. (2008). ZmXTH1, a new xyloglucan endotransglucosylase/hydrolase in maize, affects cell wall structure and composition in *Arabidopsis thaliana*. J. Exp. Bot.

